# A 3D microvascular network model to study the impact of hypoxia on the extravasation potential of breast cell lines

**DOI:** 10.1038/s41598-018-36381-5

**Published:** 2018-12-18

**Authors:** Jiho Song, Agnès Miermont, Chwee Teck Lim, Roger D. Kamm

**Affiliations:** 1 BioSystems and Micromechanics, IRG, Singapore-MIT Alliance for Research and Technology, Singapore, 138602 Singapore; 2 Department of Biological Engineering and Department of Mechanical Engineering, Massachusetts Institute of Technology, Massachusetts, USA; 3 Department of Biomedical Engineering, National University of Singapore, Singapore, 117576 Singapore; 4 Mechanobiology Institute, National University of Singapore, Singapore, 117411 Singapore; 5 Biomedical Institute of Global Health Research and Technology, National University of Singapore, Singapore, 117599 Singapore

## Abstract

Hypoxia is a common feature of the tumor microenvironment. Accumulating evidence has demonstrated hypoxia to be an important trigger of tumor cell invasion or metastasizes *via* hypoxia-signaling cascades, including hypoxia-inducible factors (HIFs). Microfluidic model can be a reliable *in vitro* tool for systematically interrogating individual factors and their accompanying downstream effects, which may otherwise be difficult to study in complex tumor tissues. Here, we used an *in vitro* model of microvascular networks in a microfluidic chip to measure the extravasation potential of breast cell lines subjected to different oxygen conditions. Through the use of HIF-1α knock-down cell lines, we also validated the importance of HIF-1α in the transmigration ability of human breast cell lines. Three human breast cell lines derived from human breast tissues (MCF10A, MCF-7 and MDA-MB-231) were used in this study to evaluate the role of hypoxia in promoting metastasis at different stages of cancer progression. Under hypoxic conditions, HIF-1α protein level was increased, and coincided with changes in cell morphology, viability and an elevated metastatic potential. These changes were accompanied by an increase in the rate of extravasation compared to normoxia (21% O_2_). siRNA knockdown of HIF-1α in hypoxic tumors significantly decreased the extravasation rates of all the cell lines tested and may have an effect on the function of metastatic and apoptotic-related cellular processes.

## Introduction

Hypoxia within the tumor microenvironment plays a central role in regulating breast cancer progression, metastasis, and patient mortality^1–4^. Hypoxia-inducible factors (HIFs) are a family of transcription factors that regulate the expression of hypoxia-inducible genes in response to reductions in oxygen concentration. HIFs are heterodimeric complexes composed of two subunits, an α-subunit whose level increases during hypoxia and a β-subunit that is constitutively expressed^1^. HIFs regulate over 1000 gene products by binding hypoxia response elements (HREs) at target gene loci^5,6^. More precisely, many cellular processes controlled by HIFs are linked to cancer development such as angiogenesis, metabolic reprogramming, epithelial-mesenchymal transition (EMT), invasion, and metastasis^7–11^. HIF-1 and HIF-2 are closely related key transcriptional regulators of the hypoxic response. HIF-2α is low or absent from the more aggressive cell lines. However, HIF-1α, which is regulated by a proline hydroxylase^4^, has been described to control many important steps of the metastatic process and promotes an aggressive cancer phenotype^6,12–15^. Indeed, overexpression of HIF-1α has been confirmed in many primary tumor biopsies, and is associated with resistance to therapy, and poor outcomes^16–19^.

Breast cancer is the most commonly diagnosed cancer and the second leading cause of cancer death among women^20^. Early detection of relapsed and metastatic disease has been a primary focus of ongoing research^21^. Hypoxia is present in over 90% of solid tumors, and the mean partial pressure of oxygen (PO_2_) is 10 mm Hg in breast cancer as compared to 65 mm Hg in normal human breast tissue^6^. PO_2_ values less than 10 mm Hg have been associated with an increased risk of metastasis and mortality^3^. Using HIF-1α as a marker for hypoxia^14^, it has been observed that approximately 25 mm Hg^22,23^ hypoxic tumors are associated with a more aggressive phenotype^24^, increased risk of metastasis^5^, increased resistance to radiotherapy and chemotherapy^25^, and induced cancer immune suppression^26–28^.

Cancer metastasis is a complex and dynamic multi-step process^29–31^. During metastasis, many interactions occur among tumor cells and their surrounding microenvironment, and these interactions can have far reaching effects on the intrinsic metastatic potential of the cancer cells. *In vitro* models for studying cancer metastasis have thus relied heavily on the use of simple assay systems that do not allow expression of the full spectrum of interactions and events that occur during metastasis. Among conventional models, the Boyden chamber transwell assay is the most commonly used in the study of tumor cell invasiveness, in which cells migrate by chemotaxis from an upper environment toward a bottom chamber by crossing a porous membrane^32,33^. Recent advances in microfabrication technologies and biomaterials have allowed for the development of *in vitro* platforms that recapitulate physiologically relevant cellular processes of cancer progression. In past years, many groups have developed 2D endothelial monolayer and 3D microvascular models to investigate tumor angiogenesis^34–36^, intravasation^24,25,37^, role of interstitial flow^38–40^, cancer cell migration^41,42^, and extravasation^43–45^. The engineered 3D microvascular network system developed by our group is a robust experimental model for creating readily perfusable blood vessels *in situ* imaging and quantification of the critical metrics of cell-cell interactions or cancer cell invasiveness^46–49^. Using such devices allowed a better description of the different stages of trans-migration. The first step consists in cell penetration through the endothelial barrier by extending filopodial protrusions. Protrusions will then increase and branch out while the remaining body on the apical side of the lumen maintains its sphericity. β1 integrin activation facilitates protrusion maintenance through focal adhesion proteins (e.g., vinculin) and F-actin recruitment to the tips of protrusions; actomyosin-mediated contractions pull the remaining spherical cell body past the endothelial barrier and cells undergo shape changes as they adopt a final spread morphology^43,45,50^.

Identification of key alterations that occur under hypoxic conditions is critical to elucidating mechanisms that promote metastasis. In this study, we investigated the impact of hypoxia on cancer progression using a panel of breast cell lines with different degrees of malignancy, namely the non-malignant breast cell line MCF-10A and the two breast carcinoma cell lines MCF-7 and MDA-MB-231. Considering multiple cell lines permit a more exhaustive study on how hypoxia affects both MCF-10A and cancer cell lines based on their stages of cancer progression. Cell proliferation, viability and invasiveness potential were each studied under long-term hypoxia (i.e. three to five days), therefore amplifying the effect of low oxygen on the different types of breast cell lines. Finally, a 3D microvascular network was used to unravel the role of hypoxia and HIF-1α in the extravasation potential of breast cell lines. Using our *in vitro* 3D model, we can address such important issues that are otherwise difficult to investigate in the clinic. Continued studies which examine specific mechanisms of the role of hypoxia in metastasis are clearly warranted and may likely lead to new and innovative therapeutic strategies to block metastasis. To the best of our knowledge, this is the first study evaluating the role of HIF-1α in the extravasation of human breast epithelial and cancer cell lines in 3D microvasculature. Our data provide evidence that hypoxia and HIF-1α have an essential role in promoting aggressive behavior and extravasation potential regardless of the level of malignancy of the cell lines.

## Results

### Effect of hypoxia on proliferation and cell viability

**Figure 1:**
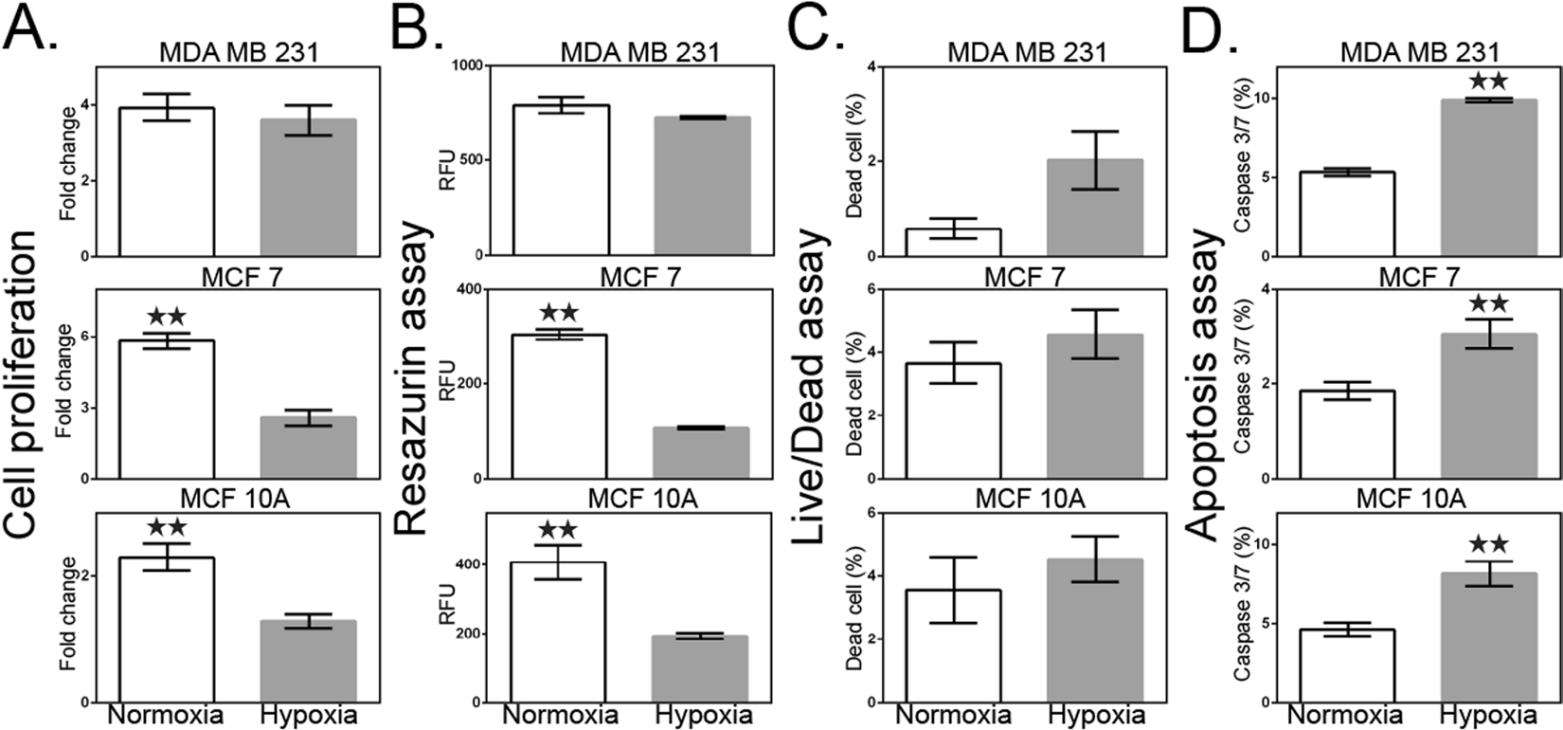
Effect of hypoxia on cell proliferation and viability. (**A**) Rate of proliferation defined by the ratio of the number of viable cells at day 5 to the number of viable cells at day 1. (**B**) Metabolism assay using resazurin dye. Cellular metabolic activity is measured as relative fluorescence units (RFU). (**C**) Flow cytometric analysis of live/dead fixable aqua dead cell stain using the SYTOX assay. (**D**) CellEvent Caspase-3/7 Green was used to measure an early indicator of apoptosis of cells destined for cell death. White bar represents normoxia. Grey bar represents hypoxia. Data are presented as the mean ± SEM of triplicate samples. **p < 0.01 versus normoxia-treated cells.

To investigate the impact of hypoxia on cell proliferation and viability, four cell-based assays were performed on the three human breast cell lines exposed to either hypoxia or normoxia for 5 days. Cell proliferation was first measured by counting the viable cells at different days. After 5 days in culture, we observed that the hypoxia groups of MCF-7 and MCF-10A cells had significantly lower number of cells compared to normoxic groups. The MDA-MB-231 hypoxic group presents also a lower number of cells compared to normoxia; however the difference for this cell line is not significant (Fig. 1A, n = 4).

In order to strengthen our observations, we investigated the metabolic activity of breast cell lines under both hypoxic and normoxic conditions. Using resazurin dye as a metabolic activity marker of cells, we confirmed that hypoxia had little effect on MDA-MB-231 cells, while MCF-10A and MCF-7 cells exhibited a significantly reduced metabolic activity (Fig. 1B, n = 3). Using the SYTOX live/dead assay, we observed a similar trend (Fig. 1C, n = 3). Indeed, the number of dead cells for each breast cell line was higher in hypoxia compared to normoxia. However, the viability difference between conditions was not significant, in contrast to the significance of the resazurin and cell count results for MCF-10A and MCF-7 cells.

In order to look more deeply into the mechanism of cell death, and notably if cells are undergoing apoptosis, the activity of Caspase-3/7 was investigated. We observed that the percentage of apoptotic cells was significantly increased in hypoxia compared to normoxia, and this for all cell lines investigated (Fig. 1D, *p* < 0.01; n = 5). This is supported by evidence showing the impact of hypoxia in promoting the apoptotic pathway^51^.

### Hypoxia induces changes in cell morphology and gene expression associated with invasive phenotype

It is known that hypoxia induces morphological changes linked to a more invasive behavior^52^. In order to confirm the impact of hypoxia on cell shape and structure, the three breast cell lines (MDA-MB-231, MCF-7 and MCF-10A) were exposed to hypoxic conditions for 72 h, and their morphology compared to cells incubated in normal oxygen level (Fig. S1). Control cells under normoxic conditions showed robust cellular junctions with a cobblestone-like, epithelial appearance. Conversely, under hypoxia, all cells appeared flattened and fibroblast-like, characterized by many cytoplasmic projections and loss of tight cell-cell junctions typical of an invasive phenotype (Fig. S2). Loss of epithelial E-cadherin and gain of mesenchymal vimentin often correlate with increased invasive behaviour and are used as markers of cancer progression.

**Figure 2:**
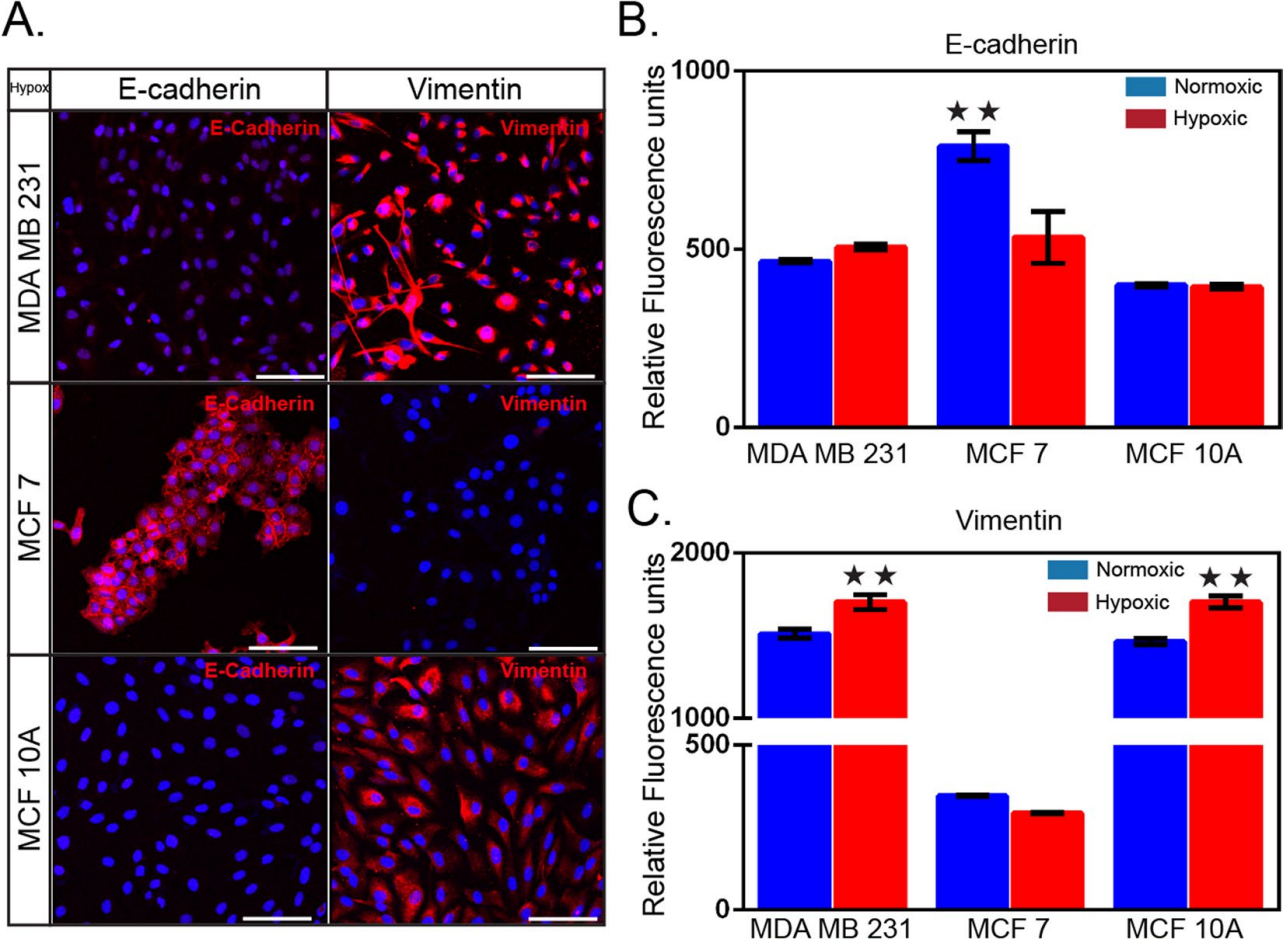
Changes in cell morphology and gene expression under hypoxia. (**A**) Immunofluorescent staining (red) of the epithelial marker E-cadherin (HECD-1) and the mesenchymal marker vimentin (V9). Three human breast cell lines, MDA-MB-231, MCF-7, and MCF-10A cells were cultured in hypoxia for 72 h prior to staining. The secondary antibody is rabbit anti-mouse labelled with AlexaFluor 594. Cell nuclei were stained with DAPI. Relative fluorescence intensity of (**B**) E-Cadherin and (**C**) vimentin. Cells were cultured in normoxic and hypoxic conditions for 72 hours prior to the staining. Mean ± S.E.M. data are shown (n = 3); **p < 0.01 (Student’s *t* test). Scale bar is 100 µm.

In order to investigate whether the observed morphological switches correlate with enhanced migratory properties, we investigated the expression of vimentin and E-cadherin in hypoxia and normoxia conditions using immunofluorescence. As shown in Fig. 2A–C, both MDA-MB-231 and MCF-10A showed similar profiles of expression in both conditions consisting in high vimentin expression, and low level of E-cadherin. This observation was expected since both cell lines present features of mesenchymal phenotype. In contrast, MCF-7 present an epithelial profile in both oxygen conditions, characterized by high levels of E-cadherin while vimentin expression remains low (Fig. 2A–C). However, when comparing hypoxia over normoxia condition, we observed a significant increase in vimentin expression in both MDA-MB-231 and MCF-10A cells (Fig. 2C) while no significant changes could be detected for E-cadherin expression. For MCF-7 cells, we observed the opposite trend, i.e. a 1.5-fold decrease in E-cadherin expression under hypoxic conditions while no significant changes in vimentin expression could be detected. For MCF-7 in normoxic conditions, E-cadherin localized at the plasma membrane and the cells formed small islands (Fig. 2A). In contrast, under hypoxia, we observed a loss of cell polarity as well as a decrease in E-cadherin expression (Figs 2B and S1-2). MDA-MB-231 and MCF-10A cells did not form clusters in either condition but still present a decrease in cell-to-cell polarity under hypoxic conditions (Figs 2A and S1). As the loss of E-cadherin and/or increase in vimentin expression were expected features of cancer progression, those results suggested that hypoxia triggers - to a certain extend and for the three cell lines tested – changes in expression associated with increased invasiveness.

### HIF-1α expression during hypoxia and validation of HIF-1α knockdown cell lines

**Figure 3:**
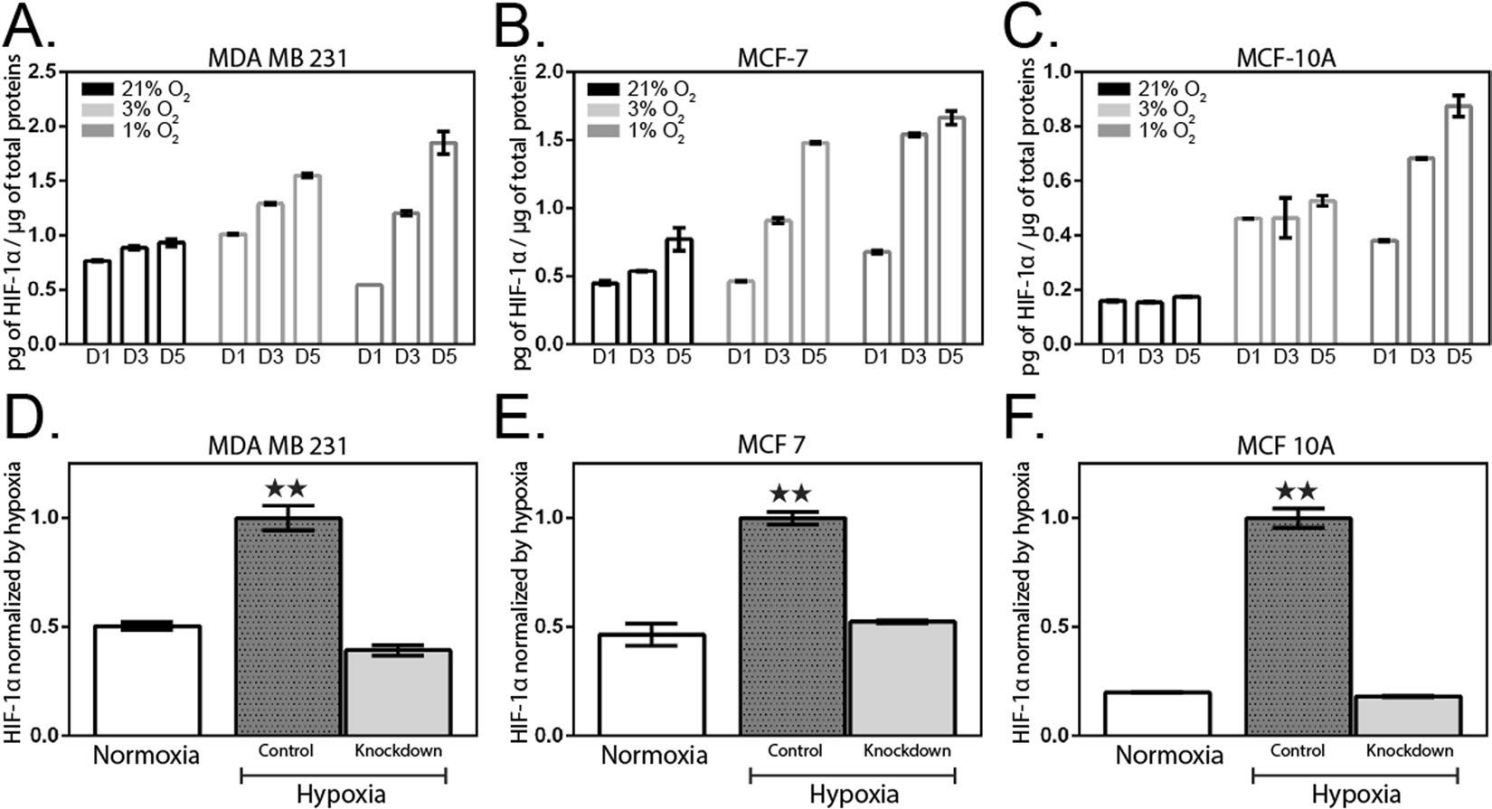
HIF-1α measurement in hypoxic-induced cell lines. Three breast cell lines; (**A**) MDA-MB-231, (**B**) MCF-7, and (**C**) MCF-10A, were harvested after 1-, 3-, 5-days under normoxic (21% O_2_) and hypoxic (1% and 3% O_2_) conditions. Normalized HIF-1α levels over total protein levels. (**D**–**F**) HIF-1α knock-down using siRNA. Corresponding bar graphs represent relative HIF-1α normalized to the respective control cells in hypoxic condition. Data are presented as the mean ± SEM of duplicate independent samples. **p < 0.01 versus hypoxia-treated cells; n = 2 due to consistent datasets.

HIF-1α is a key control factor in the transcriptional response to oxygen. In order to obtain a quantitative description of the impact of hypoxia on HIF-1α expression, we applied various oxygen conditions (21%, 3%, and 1%) with constant CO_2_ and temperature to the three human breast cell lines previously tested (Fig. 3). HIF-1α protein level was analyzed up to five days using an enzyme-linked immunosorbent assay (ELISA). We observed increased levels of HIF-1α in hypoxia compared to normoxia for all cell lines tested, which is consistent with previous studies^53,54^. In addition, the level of HIF-1α increased with exposure time and peaked on day 5 for all cell lines (Fig. 3A–C). In hypoxic conditions (1% and 3% O_2_) at day 5, HIF-1α level was higher for all cell lines compared to normoxic condition (21% O_2_). Same observations have been made for shorter induction times (day 1 and 3) except for MDA-MB-231 cells incubated in 1% O_2_ at day 1 for which HIF-1α protein level is lower than in normoxia. The difference in HIF-1α level at day 5 between hypoxia 1% O_2_ and normoxia 21% O_2_ is significant for MDA-MB-231 (1.67-fold increased), MCF-7 (2.1-fold increase) and MCF-10A (5.0-fold increase). Therefore, the protein level of HIF-1α depends on hypoxic conditions; i.e. oxygen level and incubation time, but is independent on cell phenotype.

In order to confirm those observations, we used a siRNA-mediated approach to knockdown HIF-1α in the three breast cell lines previously cultured under hypoxia for several days. Such approach was used to determine whether the reduction in HIF-1α would result in a loss of the previously observed phenotypes. Under hypoxic condition, HIF-1α knockdown cells present a significant reduction of HIF-1α protein levels for MDA-MB-231 (0.39 ± 0.02; 61%), MCF-7 (0.52 ± 0.01; 48%) and MCF-10A (0.16 ± 0.00; 84%) compared to the control group (HIF-1α normalized to control cells in hypoxia, Fig. 3D–F, respectively). Hypoxic HIF-1α knockdown cells present minimal difference or nearly the same values than control cells in normoxia (Fig. 3D–F).

### Hypoxia promotes human breast cell extravasation

**Figure 4:**
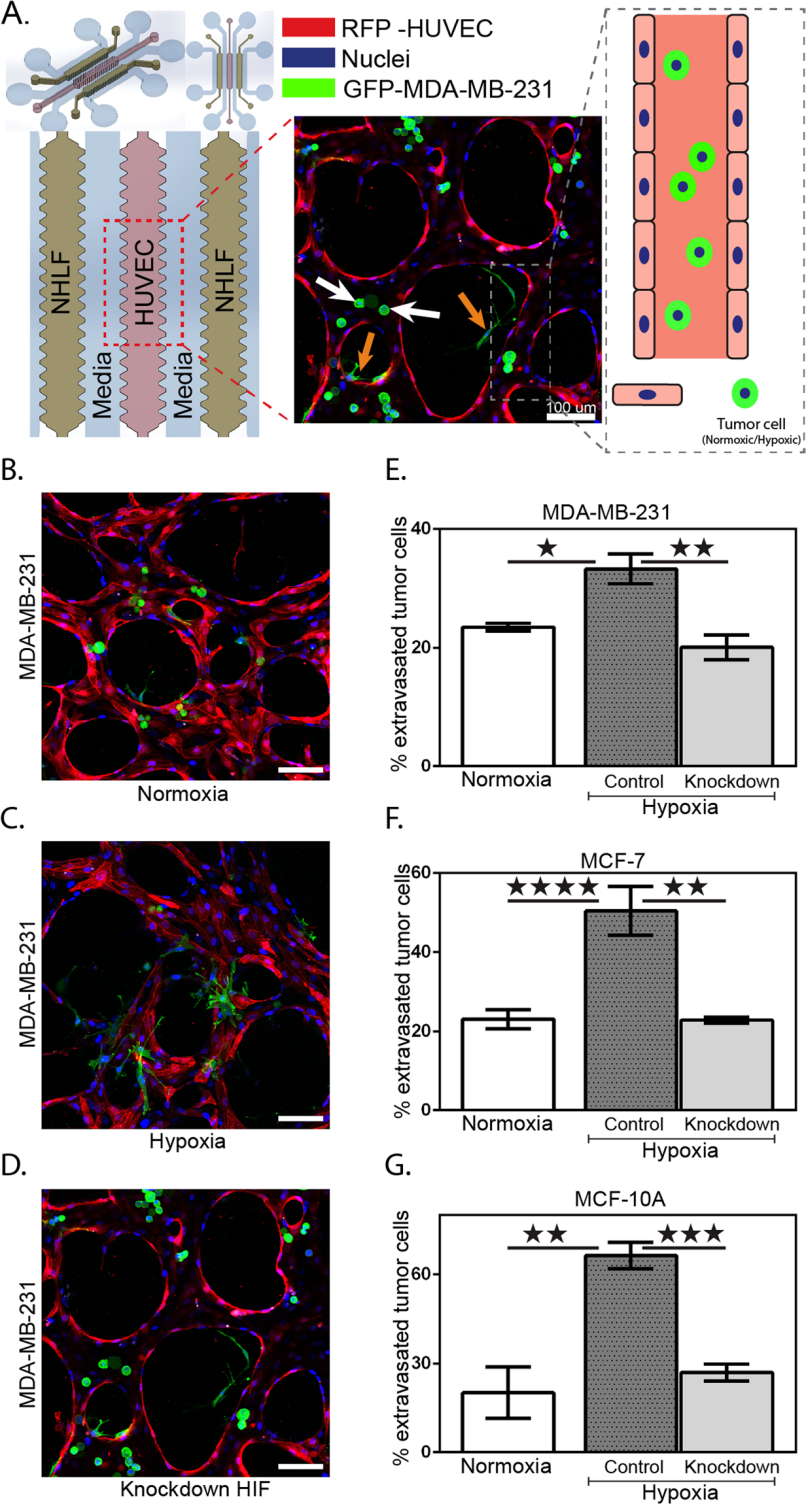
*In vitro* extravasation model of microvascular network system. (**A**) Schematic illustration of the device structure with three-gel channels; HUVECs seeded in middle channel, and along the sides NHLF. HUVECs form vasculature and NHLF are stabilizers. Human breast cells were introduced into the media channel to reach the vasculature network. The fluorescent image shows the vascular network (RFP-HUVEC) with GFP-MDA-MB-231 cells. Orange arrows indicate representative extravasated cells, whereas white arrows indicate representative non-extravasated cells. Images of MDA-MB-231 cells introduced into a 3D microvascular network with cells cultured in (**B**) normoxia, (**C**) hypoxia, and (**D**) HIF-1α knockdown showing extravasation. (**E**–**G**) Graphs of extravasation rates for three breast cell lines in different conditions. Scale bar in all panels, 100 µm. Mean ± S.E.M. data are shown. P values were calculated by one-way ANOVA with Bonferroni post-test. *p < 0.05, **p < 0.01, ***p < 0.001. n = 5 for each condition.

Next, we examined the effect of hypoxia on human breast cell extravasation using a functional microvascular network generated in a microfluidic platform^45,55^. The cultured HUVECs start forming microvascular networks within 24 hours and perfusable vessels with patent lumens formed after 3–4 days depending on the seeding density (Fig. S3). Then, a pressure gradient was established across the vascularized compartment and human breast cells, pre-treated under either normoxic or hypoxic conditions, were suspended in the medium and introduced in the vessels where they arrested and began extravasating across the endothelial cell barrier into the hydrogel (Fig. 4A).

Cells that either adhered to the endothelium or became physically trapped inside the small vessels were imaged after 6 hours incubation to determine the rates of extravasation (Figs 4B–D and S4). We first performed a control experiment where we measured the extravasation rate of cells pre-treated under the normoxic condition (n = 5). We observed that the average rate was relatively similar among the different cell lines (Fig. 4E–G). We then explored the impact of pre-treatment with hypoxia on the extravasation rate of the three breast cell lines. Extravasation rates were significantly higher for all three cell lines compared with the normoxic cells (Fig. 4E–G). The average extravasation rate was 33.28 ± 2.49% for MDA-MB-231, 50.45 ± 6.15% for MCF-7, and 66.41 ± 4.45% for MCF-10A, or an increase of 1.5- to 3.5-fold compared to normoxic cells (Fig. 4E–G; Hypoxic). Similarly, we tested the transmigration ability of the knockdown HIF-1α breast cells after hypoxia pre-treatment. The extravasation rates of the three knockdown HIF-1α cell lines were significantly lower than the wild-type cells in hypoxic conditions; notably, values were similar to the extravasation rates of wild-type cells cultured in normoxic conditions.

## Discussion

The control of O_2_, CO_2_, and temperature is essential to maintain cell homeostasis, and small deviations in these parameters can affect gene expression and cell phenotype. While physiological *in vivo* oxygen concentrations can range from 1% to 15%, most cell cultures are maintained at 21% O_2_. Therefore, the use of hypoxic culture conditions may be more meaningful for research aimed at investigating natural phenomena. Hypoxic conditions are found in most tumors and are known to promote tumor invasion and metastasis via multiple mechanisms^8,10^. For instance, the high proliferation rate of solid tumors cause some of the tumor cells to become progressively distant from vasculature and they soon encounter an environment deficient in oxygen. 90% of tumors are described as hypoxic and are associated with aggressive phenotype, increasing the risk of metastasis and resistance to therapy^56^.

In this work, a panel of breast cell lines with different degrees of malignancy was used to study how hypoxia affect cells depending on their stages of cancer progression. The breast cell lines used consisted of the immortalized breast cell line MCF-10A, the tumorigenic but non-invasive cell line MCF-7 and the tumorigenic and invasive MDA-MB-231 cell line. Previous studies using the three aforementioned cell lines mainly focused on one aspect of hypoxia, either investigating a specific signalling pathway^57–59^, or a molecular factor involved in cancer progression^60,61^ but none interrogated simultaneously impact hypoxia on proliferation, cell death, HIF-1α expression and extravasation.

We first investigated the impact of hypoxia on cell proliferation and viability. Previous studies showed that hypoxia induces either no change or an increase in breast cancer cell proliferation, and no change or a decrease in apoptosis^54,62–64^. On the contrary, the breast cell line MCF-10A has been observed to decrease proliferation, and induce apoptosis under hypoxia^65^. Therefore, different responses were described depending on the malignancy of the breast cell lines, with higher sensitivity for non-tumorigenic breast cell types to hypoxia. Moreover, hypoxia was never induced more than 72 h, while longer time might have been needed for oxygen to be a limiting factor. One novel aspect of our study was to culture the cells for 5 days in low oxygen, therefore amplifying the effect of hypoxia on different types of breast cell lines. Surprisingly, we observed in those conditions that proliferation was significantly decreased in both MCF-7 and MCF-10A; while MDA-MB-231 showed only a mild, not significant reduction. Considering hypoxia-induced cell death, we observed a significant increase in apoptosis for all three cell lines. Therefore, long-term hypoxia seems to reduce the differences previously observed with short durations of hypoxia among cell lines with different degrees of malignancy in that metastatic cell lines show, or begin to show, similar sensitivity to that of non-metastatic breast cells. The impact of hypoxia on cell death depends on different factors such as cell type and time of exposure. In general, if nutrients are accessible to the cells, apoptosis, an energy dependent cell death, can be triggered. Different mechanisms are involved, including the release of cytochrome C from mitochondria^66^, the generation of reactive oxygen species (ROS) and the activation of c-Jun NH2-terminal kinase (JNK)^67^. However, in the absence of ATP, hypoxic tumor cells are unable to enter the apoptotic cascade and cells undergo necrosis, often observed in the central core of solid tumors^51^. In this work, the breast cell lines are cultured in conventional monolayer conditions with sufficient access to nutrient at all time, and are therefore able to initiate apoptosis.

Next, we investigated the impact of hypoxia in promoting invasiveness through the expression of well-known markers^68^. Aggressive phenotype of metastatic cells is defined by the loss of epithelial characteristics^42,69^, notably E-cadherin involved in cell-cell adhesion^70^, and the increase in cell motility and expression of mesenchymal markers, such as vimentin^71^. This overall change correlates with increased migration and invasion capacity of breast cancer cells^72–74^. MDA-MB-231 is a mesenchymal-like cell line and MCF-10A, despite being a non-tumorigenic epithelial cell line, shares many features of a mesenchymal cancer cell line^75–77^. Indeed, due to vimentin positivity and low E-cadherin expression, both present post-EMT properties in normal condition (Fig. 2A–C). Culturing MDA-MB-231 and MCF-10A in hypoxic conditions triggers an increase in vinculin expression but no change in E-cadherin. The naturally low level of E-cadherin may imply that further reduction is either not possible or too energy consuming for the cell. However, the increase in vimentin expression for both cell lines could reflect a progression to a more invasive phenotype. The last cell lines used in this study, MCF-7, is classified as a luminal cell line^78^ with an epithelial phenotype. Indeed, we observed in normoxic condition high E-cadherin and low vimentin expression (Fig. 2A–C). During hypoxia, MCF-7 cells displayed a significant decrease of E-cadherin expression but no change in vimentin level could be observed. Even though the change of expression experienced by the different breast cell lines is only observed for one of the two markers analysed, we cannot disregard the fact that the modification correlates with either loss of epithelial phenotype or increased invasiveness, which are all markers of cancer progression.

As previously described, hypoxia induced the stabilization and accumulation of HIF-1α protein which is a feature of metastatic progression in various cancers^79^. The downstream target genes of HIF-1α are related to angiogenesis, cancer cell survival and invasion^4^. In addition, HIF-1α has been observed to regulate factors of many EMT regulators, such as SNAI1, twist or LOX and promotes EMT in different cell lines^80–82^. These previous studies motivated us to investigate the role of HIF-1α in metastatic progression of hypoxic cells. Our results showed that for the three cell lines tested, HIF-1α level increases in hypoxia and correlates with the percentage of oxygen and period of exposure (Fig. 3A,C). Those results are in accordance with previous studies showing a direct link between HIF-1α induction and hypoxic tumors^53^. The direct role of HIF-1α in hypoxia-induced apoptosis previously described is also consistent with our observation, showing that hypoxia triggered an increase in both HIF-1α protein level and apoptosis for each breast cell line tested.

We then investigated the role of hypoxia and HIF-1α in extravasation using our panel of breast cell lines. Previous studies have described how hypoxia increases cell motility^62^, as well as their ability to transmigrate through endothelial monolayers^83^. Most notably, Jin *et al*. investigated the role of CXCR4 expression in promoting cancer cells adhesion and transmigration through a monolayer of endothelial cells^84^. The experiment was conducted in a 2D migration assay model, and therefore fails to recapitulate the native 3D environment encountered by cancer cells. On the other hand, Zhang *et al*. used animal models by injecting hypoxic DKD cells into the tail vein of mice and analyzed lung sections one week later^11^. Despite the physiological relevance of their *in vivo* study, visualization was limited and the process of cell extravasation could not be assessed independent of the colonization step of cancer cells into the lung tissue. In this study, we used engineered 3D vascular networks developed by our group to recapitulate the process of cancer cell extravasation through the vasculature. Using this platform, we observed that extravasation rates of breast cell lines previously exposed to hypoxic conditions significantly increased compared to normoxic cells. In contrast, knock-down of HIF-1α significantly reduced extravasation rates compared to wild-type cells under the same hypoxic conditions, with rates similar to normoxia. Therefore, our results support the hypothesis that hypoxia and HIF-1α induction promotes extravasation of MCF-10A and the cancer breast cells. Interestingly, the effects of hypoxia are relatively independent of the degree of cell malignancy since HIF-1α induction, apoptosis induction and extravasation rates showed similar changes among the three breast cell lines tested. Overall this study allowed an exhaustive analysis of the impact of low oxygen supply on the general biological features and specific extravasation behaviours of three breast cell lines.

## Conclusions

In this work, we have employed a previously developed microfluidic extravasation assay consisting of a self-organized 3D microvasculature that enables the study of tumor cell extravasation. *In vivo* models often lack a high level of control while classic *in vitro* cancer models provide control, yet lack critical components of the tumor microenvironment. Thus, our 3D microvasculature model served to investigate the extravasation potential of hypoxic human breast epithelial and cancer cell lines while confirming the implication of HIF-1α in this process. Hypoxia can play an important and beneficial role in human physiology and development, as well as during cancer progression. However, the role of hypoxia and the associated increase in HIF-1α protein levels on tumor invasiveness remained unclear. In this study, we characterized, for the first time, the extravasation dynamics of three different breast cell lines, pre-conditioned by hypoxia, in high spatiotemporal resolution within an *in vitro* microvascular network. However, more studies will be needed to understand the downstream cell signaling pathways, functional consequences, target genes, and suppression of HIF-1α to develop novel treatments or to inhibit tumor progression.

## Methods

### Cell culture and treatments

HUVECs (C2519A, Lonza, MA, USA) were cultured in EGM-2 BulletKit (CC-3162, Lonza) containing EBM-2 Basal medium supplemented with EGM-2 SingleQuot Kit (2% FBS, 0.4% hFGF-2, 0.1% VEGF, 0.1% R^3^-IGF-1, 0.1% hEGF, 0.04% hydrocortisone, 0.1% ascorbic acid, 0.1% heparin, and 0.1%-GA-100) and used at passage 5. NHLF were cultured in FGM-2 Fibroblast Growth Medium BulletKit (CC-3132) which consists of Fibroblast Basal Medium supplemented with FGM-2 SingleQuot Kit (2% FBS, 0.1% hFGF-B, 0.1% insulin, and 0.1%-GA-100), until passage 5. MDA-MB-231 (HTB-26) and MCF-10A (CRL-10317) human breast cell lines were obtained from ATCC while MCF-7 (AKR-211) cell line was obtained from Cell Biolabs. The MDA-MB-231 and MCF-7 cell lines were grown in DMEM (Dulbecco’s Modified Eagle Medium) containing 10% FBS and 2% penicillin – streptomycin (PS). MCF-10A cells were grown in Mammary Epithelial Basal Medium (MEGM) supplemented with SingleQuots from the MEGM Bulletkit (Lonza, MD, USA). Each human breast cell line was cultured as described by ATCC and Cell Biolabs and were confirmed to be free of microbial and mycoplasma contamination using a mycoplasma detection kit (LT07-701; Lonza, MA, USA). 80% confluent breast cells were used, and culture medium replaced every 2 days. Cells were placed in a sealed hypoxic incubator (Panasonic’s MCO-5M) that was continuously infused with a mixture of 1% O_2_ and 5% CO_2_ at 37 °C for 5 days. Control cells were cultured simultaneously in a normoxia incubator (21% O_2_, 5% CO_2_, 37 °C; Thermo Fisher Scientific, Rochester, NY, USA).

### Fabrication of microfluidic device

The microfluidic device was fabricated with polydimethylsiloxane (PDMS, Silgard 184; Dow Chemical, MI, USA) from a silicon wafer mold produced by standard photolithography techniques. Microfabrication methods were described previously for similar devices developed by our group^43,85,86^. The microfluidic device consisted of two lateral media channels and a central gel channel of 1 mm wide and 150 µm height.

### Three-dimensional microvascular networks formation

The complex endothelial-tumor cell interactions were studied using a microfluidic model of the human microvasculature, similar to the one described previously^44,85^. The microfluidic platform consisted of three parallel gel channels with human umbilical vein endothelial cells (HUVECs) seeded in fibrin gels and cultured alongside with normal human lung fibroblasts (NHLFs) to generate a microvascular network in the middle channel (Fig. 4A). Signaling between the two cell lines helped to prevent matrix degradation and maintained stable networks for a maximum of 7 days. Gel regions separated from the medium channels by trapezoidal post arrays allowing gas exchange and delivery of nutrients. Functional microvascular networks usually contain a stably formed lumen of diameter ranging between 10 and 100 µm.

HUVECs were suspended at a concentration of 12 × 10^6^ cells/mL in a solution of endothelial growth medium (EGM-2, Lonza, MA, USA) and thrombin (1 U/mL, Sigma). The cell suspension was then mixed in a 1-to-1 ratio with 20 µL fibrinogen solution (6 mg/mL) to achieve a final concentration of 6 × 10^6^ cells/mL and immediately injected into the central gel channel (Fig. 4A). NHLF cells were mixed with fibrinogen solution to reach a final cell density of 2 × 10^6^ cells/mL and injected into the two lateral gel channels. The devices were incubated within a humidity chamber at room temperature for 20 min to allow gel polymerization before filling the media channels with EGM-2. Media was replenished every 24 h for the duration of the experiment (4 days). The devices were then maintained in a humidified incubator at 37 °C and 5% CO_2_. Under these conditions, a perfusable microvasculature formed after ~4 days.

### Human breast cells perfused in three-dimensional microvascular device

Human breast cell lines were cultured under normoxic or hypoxic conditions for 5 days in EGM-2. The cell lines were then trypsinized and resuspended at a concentration of 4 × 10^5^ cells/mL in EGM-2. Breast cell lines were introduced into the microvascular networks on day 4 of device culture by flowing a volume of 40 µL of the cell suspension through the vascular networks under a pressure drop of 5.2 mm H_2_O (Fig. S3). The device was then placed at 37 °C for 30 min for stabilization. The media from the side channels were replaced to remove the excess of human breast cells. The device was once again incubated at 37 °C in a humidity chamber for 6 h, allowing for cell extravasation and invasion.

### Proliferation and cell viability assays

All experiments were conducted using the three breast cell lines. To ensure consistency among experiments, passage 3 to 6 were used in the study and the cells were cultured for 5 days under normoxic and hypoxic conditions. The same density of each cell line was seeded in culture plates and maintained at 1% and 21% O_2_. Cells were almost fully attached 2 h after seeding. The medium was replenished 24 h after seeding and every 2 days thereafter.

Cell proliferation was measured by counting the number of viable cells at different days using a trypan blue exclusion test. For that, cells were plated at 1 × 10^5^ cells per well in six-well cell culture plates. The cells were then harvested by trypsinization and centrifugation at 500 × g for 5 minutes. Cells were resuspended in trypan blue solution (Sigma) and viable cells were counted (BioRad; TC20). The ratio of cell number at day 5 to the cell number at day 1 was finally calculated.

PrestoBlue (Invitrogen; A13261) is a resazurin-based solution for rapidly quantifying the metabolic active cells, providing a metric of cell viability. The cells were seeded in 96-well plates for 5 days at 37 °C in the appropriate medium and oxygen condition. The reagent was then added directly to the cells and incubated for 10 min at 37 °C. The metabolic rates were measured by the amount of fluorescence using a plate reader (Tecan, Infinite M200 Pro).

Following, a quantitative single-step dead cell indicator, SYTOX Green Dead Cell Stain (Invitrogen; S34860), was used to identify the dead population using flow cytometry. The cells were cultured in a six-well plate for 5 days at 37 °C. The live cell population was labelled using NucBlue Live ReadyProbes Reagent (Invitrogen, R37605) for 15 min prior to SYTOX Green addition. SYTOX Green was then prepared (30 µM) in FACS buffer; PBS, 2 mM EDTA, 5% FBS, 5% human serum, 0.1% sodium azide (Merck, 1.06688.0100) and added to cells for 20 min at 4 °C, protected from light. To ensure proper staining, the solution was washed 3 times with phosphate-free buffer. The cells were then fixed with 4% paraformaldehyde (PFA) for 15 min. Flow cytometry was then performed using LSRII Flow cytometer (BD Biosciences), and data were analysed using the BD FACS Diva software (BD Biosciences).

Caspase-3/7 Green Ready Probes (Invitrogen; R37111) was used for detection of active caspase-3 or caspase-7 in cells undergoing apoptosis. Cells were seeded in 6-well plates and incubated in different oxygen conditions (1% or 21% O_2_) at 37 °C for 5 days. Prior to addition of the probes, NucBlue Live ReadyProbes Reagent was used for live cell detection. Caspase-3/7 Green Ready reagent was added to cells (2 drops/mL) and allowed 30 min incubation at 37 °C. Cells were then fixed using 4% paraformaldehyde (PFA) for 15 min. Cells were imaged using a confocal microscope (Olympus FV-1200) with a 10x objective. Apoptotic versus non-apoptotic cells were then counted using Imaris software. All the experiments were conducted in triplicate.

### HIF-1α ELISA

Concentration of HIF-1 α protein in cell culture lysates was quantified using the quantitative sandwich ELISA immunoassay (Invitrogen, HIF-1 α ELISA Kit; EHIF1A). HIF-1α standards and samples were captured by a polyclonal HIF-1α antibody on the pre-coated plate. Detection was then performed using a biotinylated monoclonal HIF-1α antibody reactive to epitopes other than the capture antibody. All reagents, samples and standards were prepared as instructed in the manual. Prior to ELISA, the cells were treated with different levels of oxygen as previously described. The cell lysate was collected in cultured adherent cells to approximately 80% confluence on T25 cell culture flask (Invitrogen). The cells were then scraped using a cell scraper and the lysate transferred to a 15 mL conical tube. The Bradford protein assay was used to measure the concentration of total protein for each sample (Invitrogen, 23246). All the experiments were conducted in triplicate.

### HIF-1α siRNA Transfection

Prior to transfection, breast cell lines were first cultured in hypoxia for 5 days and transfected with siRNA against HIF-1α for 48 h. 30 pmol of HIF-1α siRNA (Invitrogen, S6539) was used in 6-well plates and transfected to breast cells using Lipofectamine (Invitrogen) according to the manufacturer’s instructions. At 48 h post transfection, the medium of the transfected cells was replaced with fresh culture medium and let to incubate for 24 h at 37 °C prior to HIF-1α ELISA experiment. The knockdown efficiency was determined by ELISA analysis.

### Immunofluorescence

All devices were washed with PBS (Invitrogen), fixed with 4% paraformaldehyde (PFA) for 15 min and permeabilized with 0.1% Triton-X 100 solution for 10 min at room temperature. Samples were treated with 5% BSA +3% (wt/vol) goat serum solution for at least 1 h at 4 °C. After blocking, samples were incubated overnight at 4 °C with E-cadherin mouse monoclonal antibody HECD-1 (131700, Invitrogen) and vimentin mouse monoclonal antibody V9 (MA511883, Invitrogen) at a ratio of 1:100. Samples were then incubated for 2 h at 4 °C with the AlexaFluor 594 rabbit anti-mouse secondary antibody (1:200, Invitrogen). All images were captured on an inverted confocal microscope (Olympus FV-1200) and further processed with Imaris software (Bitplane Scientific Software).

### Extravasation acquisition and analysis

To quantify the extravasation events, the microvascular networks were fixed at the final time point to visualize the position of breast cells relative to the endothelial barrier. Cell nuclei were then stained with DAPI (1: 1000) in order to count the cells. Images were obtained on a FluorView FV-1200 confocal laser scanning confocal microscope (Olympus) using a 20x objective at 4 um z-steps with 800 × 800 pixel resolution. Using Imaris imaging software, the nuclei of each breast cell in a ROI was identified and marked using the spot tracker function. Only cells with nuclei found on the basal side of the endothelial barrier (either directly adjacent to or migrated away from the barrier) were counted as an extravasation event. We then calculated the extravasation efficiency as the ratio of extravasated cells to the total number of cells found inside the microvasculature by analyzing a minimum of 10 ROI per device.

### Statistical analysis

Data were presented as the mean ± SEM of at least three independent experiments. For Figs 1 to 3, comparison was performed using unpaired Student’s t-test. For Fig. 4, statistical analysis was performed with one-way ANOVA followed by Bonferroni post hoc comparisons. Statistical significance is based on **P-values < 0.01. All statistical analysis were done using GraphPad Prism (GraphPad Software, Inc., San Diego, CA).

## Electronic supplementary material

Supplementary Information
