# Supplementary material for: A 3D microvascular network model to study the impact of hypoxia on the extravasation potential of breast cell lines
Source: Sci Rep. 2018 Dec 18;8:17949. doi: 10.1038/s41598-018-36381-5 (PMC6298998; doi:10.1038/s41598-018-36381-5)
Supplement: Supplementary file 1 — Supplementary Information [file 41598_2018_36381_MOESM1_ESM.docx]

**A 3D microvascular network model to study the impact of hypoxia on the extravasation potential of breast cell lines**

Jiho Song^1,2,*^, Agnès Miermont^1^, Chwee Teck Lim^1,3,4,5^, Roger D. Kamm^1,2,*^

^1^BioSystems and Micromechanics, IRG, Singapore-MIT Alliance for Research and Technology, 138602, Singapore

^2^Department of Biological Engineering and Department of Mechanical Engineering, Massachusetts Institute of Technology, Massachusetts, USA.

^3^Department of Biomedical Engineering, National University of Singapore, 117576 Singapore.

^4^Mechanobiology Institute, National University of Singapore, 117411, Singapore

^5^Biomedical Institute of Global Health Research and Technology, National University of Singapore, 117599, Singapore.

Correspondence and requests for materials should be addressed to R.D.K. (email:rdkamm@mit.edu)

**Supplementary Information**


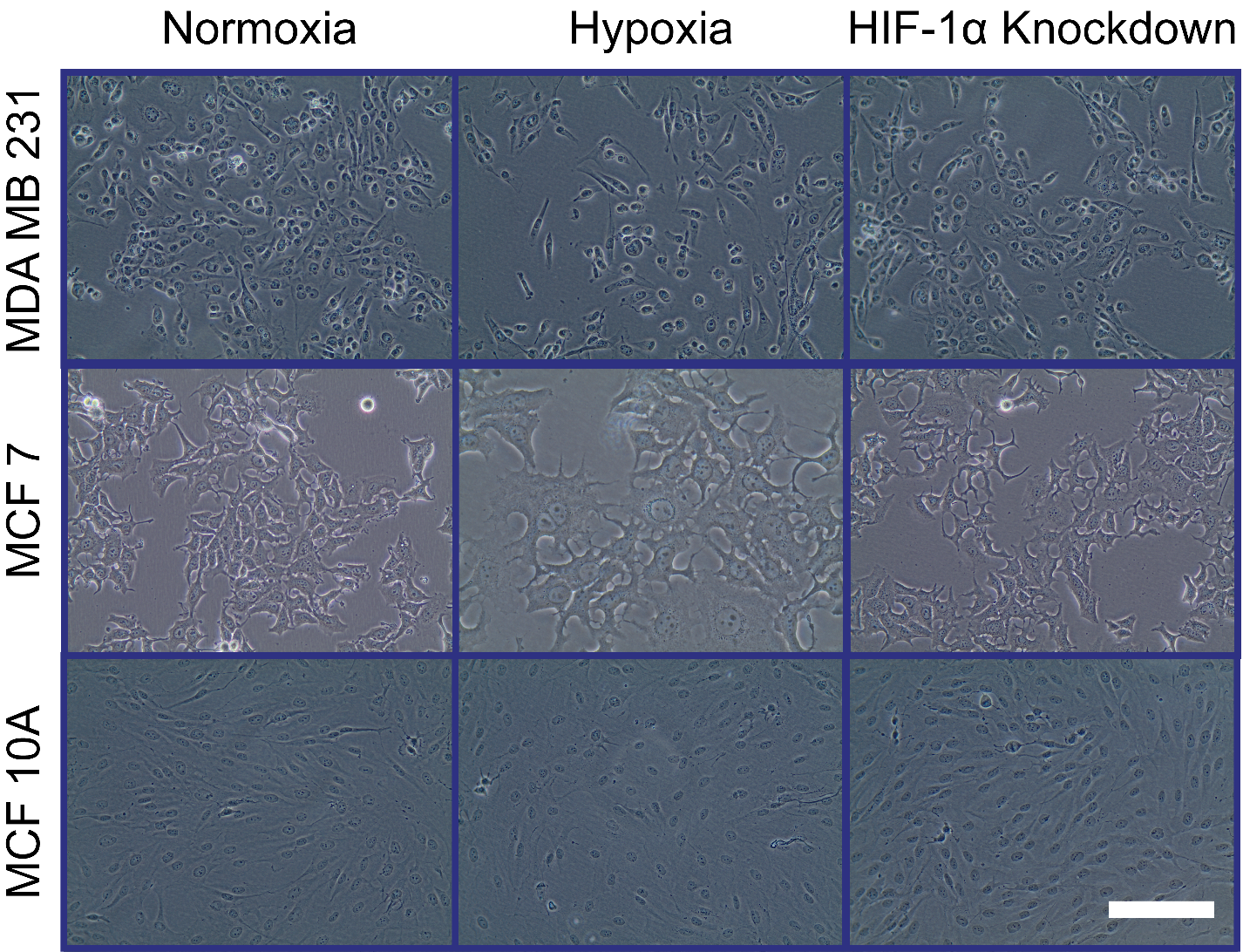


Figure S1: All cells were incubated under normoxia (21% O_2_) or hypoxia (1% O_2_) for 72 h. External cell morphology was analyzed under a phase-contrast microscope. After exposure to hypoxic conditions, cells appeared to be flatter, elongated, and ramified with long extending processes. Loss of cell-to-cell polarity was also observed after hypoxia. Scale bar = 100 um.


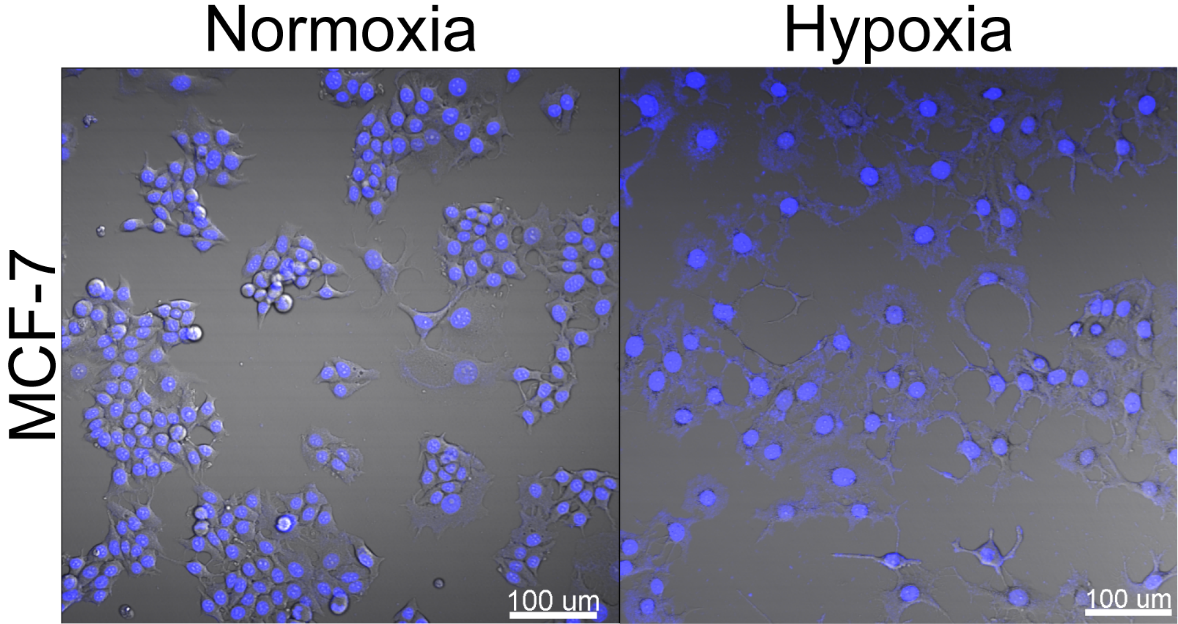


Figure S2: Phase-contrast images of MCF-7 cells after 72 h in normoxia and hypoxia conditions. Cell nucleus were stained with DAPI (blue) to distinguish cells in cluster (normoxia) and sparse (hypoxia). Scale bar = 100 um.


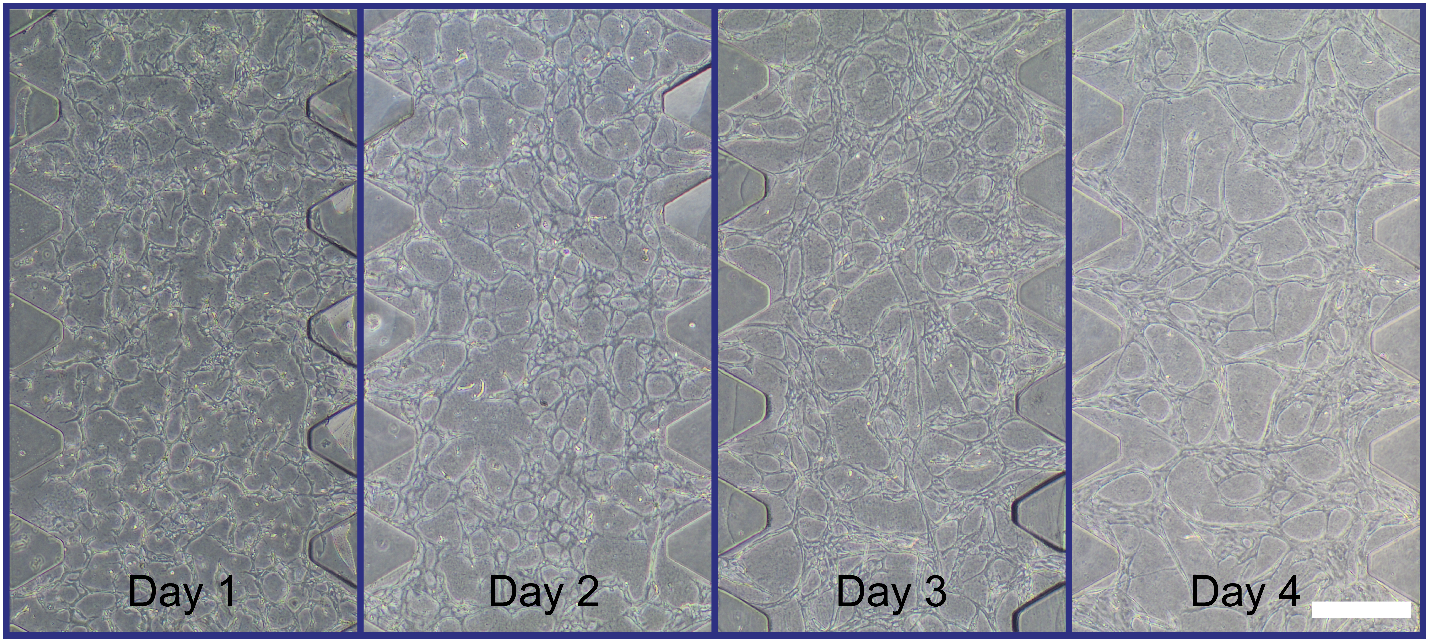


Figure S3: Time-series of a 3D microvasculature formation in the fibrin matrix. Microvasculature networks formed by endothelial cells (seeded in fibrin gels) and co-cultured alongside human lung fibroblast in separated channels. The medium was used EGM-2. The cultured HUVECs start forming microvascular networks within 24 hours and perfusable vessels with matured lumens formation occurred after 3-4 days depending on the seeding density and cell localization. Images were captured using phase contrast microscopy. Scale bar = 200 um.


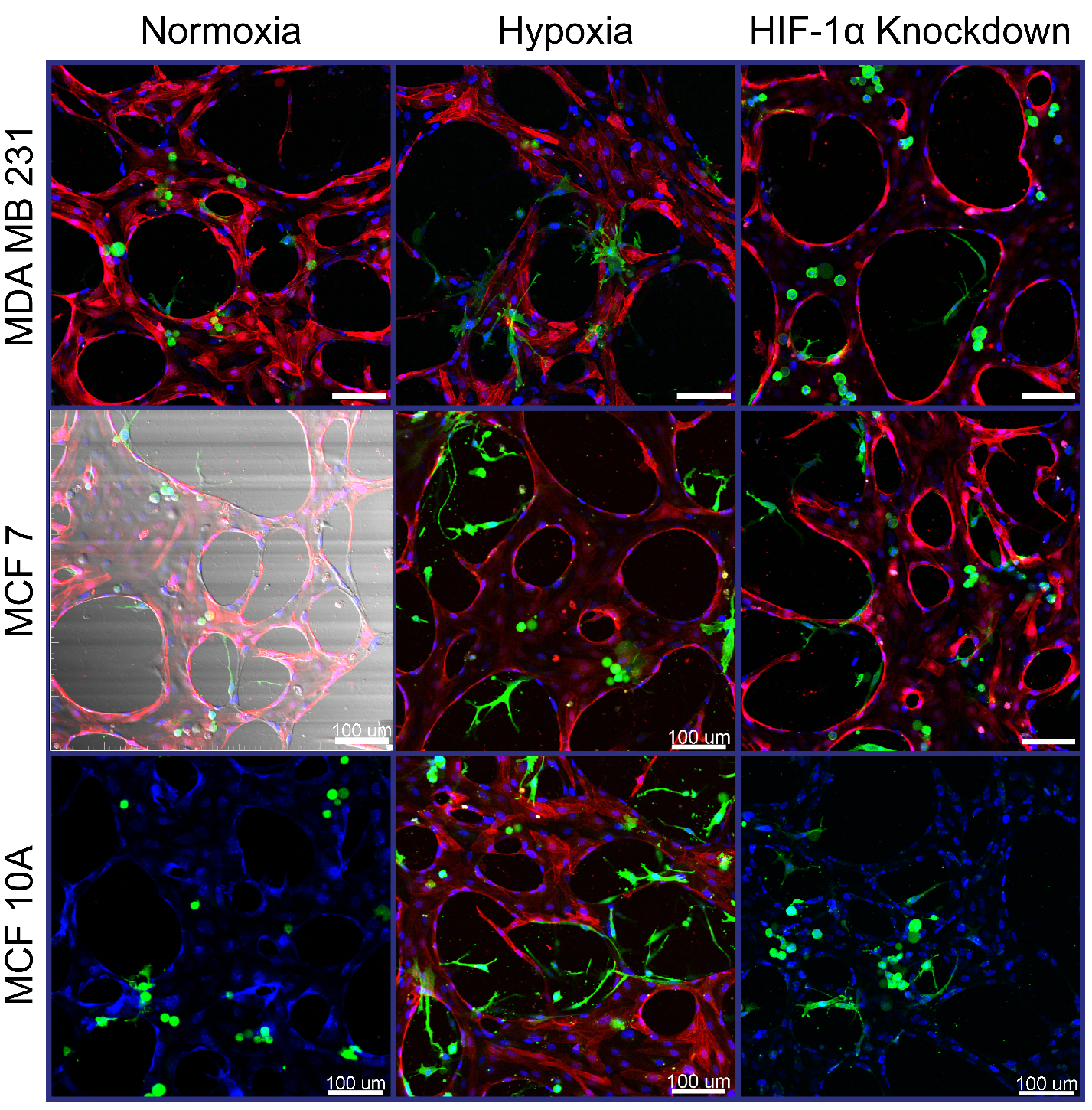


Figure S4: The fluorescent image shows the vascular network (RFP-HUVEC) with GFP-labeled breast cells. Images of breast cells introduced into a 3D microvascular network with cells cultured in (B) normoxia, (C) hypoxia, and (D) HIF-1α knockdown showing extravasation. Scale bar in all panels, 100 µm.
